# Alien Invasive Slider Turtle in Unpredicted Habitat: A Matter of Niche Shift or of Predictors Studied?

**DOI:** 10.1371/journal.pone.0007843

**Published:** 2009-11-24

**Authors:** Dennis Rödder, Sebastian Schmidtlein, Michael Veith, Stefan Lötters

**Affiliations:** 1 Biogeography Department, Trier University, Trier, Germany; 2 Sebastian Schmidtlein, Department of Geography, Bonn University, Bonn, Germany; Monash University, Australia

## Abstract

**Background:**

Species Distribution Models (SDMs) aim on the characterization of a species' ecological niche and project it into geographic space. The result is a map of the species' potential distribution, which is, for instance, helpful to predict the capability of alien invasive species. With regard to alien invasive species, recently several authors observed a mismatch between potential distributions of native and invasive ranges derived from SDMs and, as an explanation, ecological niche shift during biological invasion has been suggested. We studied the physiologically well known Slider turtle from North America which today is widely distributed over the globe and address the issue of ecological niche shift versus choice of ecological predictors used for model building, i.e., by deriving SDMs using multiple sets of climatic predictor.

**Principal Findings:**

In one SDM, predictors were used aiming to mirror the physiological limits of the Slider turtle. It was compared to numerous other models based on various sets of ecological predictors or predictors aiming at comprehensiveness. The SDM focusing on the study species' physiological limits depicts the target species' worldwide potential distribution better than any of the other approaches.

**Conclusion:**

These results suggest that a natural history-driven understanding is crucial in developing statistical models of ecological niches (as SDMs) while “comprehensive” or “standard” sets of ecological predictors may be of limited use.

## Introduction

**Figure 1:**
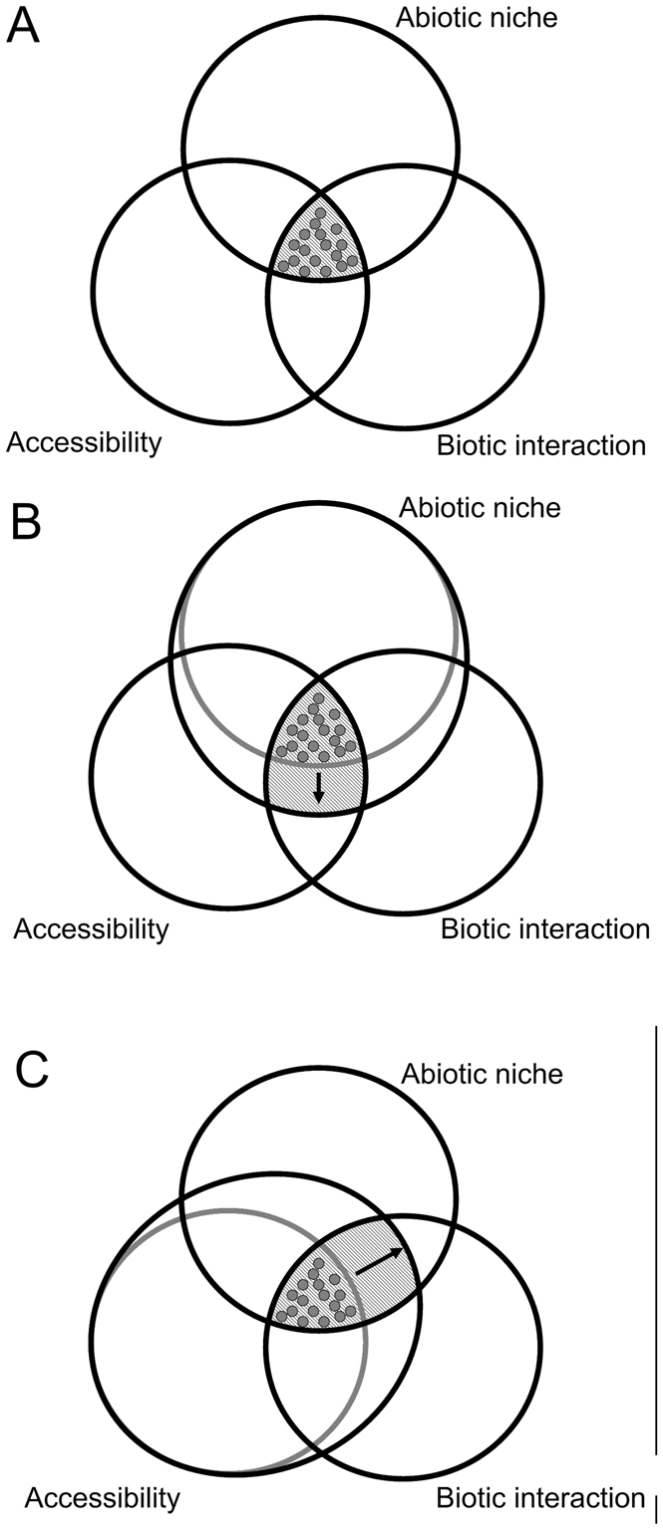
The Ecological niche concept. (A) Relationships between fundamental niche, biotic interaction and accessibility; (B) fundamental niche shift; (C) better exploitation of the fundamental niche after access into new areas [11]–[13]. Dots represent native species records of an alien invasive species from which ecological information can be used to compute Species Distribution Models to predict its potential distribution.

Alien invasive species are a concern in nature conservation as they may have a negative impact on native biodiversity [1]. To learn about the capability or risk of alien invasive plants and animals, Species Distribution Models (SDMs) are a powerful tool. A SDM characterizes the ecological niche of a species, based on ecological predictors recorded at the known distribution, and projects it into geographic space uncovering its potential distribution [2]–[5]. In recent times, there have been numerous examples in which SDMs were applied to identify areas which are suitable to certain alien invasive species. These generally aimed on climatic suitability, i.e. the species' climate envelopes [6]–[10]. In these studies, the climate envelope was understood as a part of a species' fundamental niche, which is the entirety of abiotic and biotic conditions under which it can persist [5], [11]. The portion of the fundamental niche exploited by a species is commonly limited by interactions with other species (e.g. competition, predation) as well as by spatial accessibility (e.g. through presence/absence of physical barriers) (Figure 1A) [12], [13]. It is known that fundamental niches are subject to evolution. In a recent review, it has been shown that, independent of the taxonomic group, the fundamental niche can remain stable for tens of thousands of years or that it can substantially shift within only a few generations [14]. However, there is still a considerable lack of knowledge regarding the processes triggering niche shifts.

It has been pointed out that the establishment and geographic range extension alien invasive species can provide valuable insights into ecological and evolutionary processes [15]. Indeed, some recent studies have addressed the question of rapid ecological niche shifts during invasion processes. Using SDMs, it was found that in the Spotted knapweed (*Centaurea maculosa*) the climate envelopes in its native range (western North America) differed from its invasive range in Europe [16]. Similarly, it was demonstrated in a SDM approach that Fire ants (*Solenopis invicta*) can be ascribed to climate envelopes in their invaded range (North America) from which they are absent in their native South American range [17]. These observations could represent a shift either in the fundamental (Figure 1B) or realized niches (Figure 1C). Since alien invasive species, by definition, access areas they were absent from before, the ‘new’ climate envelope might most likely simply represent a better exploitation of the existing fundamental niche (Figure 1C). To the best of our knowledge, information on the physiological limits of *Centaurea maculosa* and *Solenopis invicta* is sparse. Hence, it cannot be ruled out that the climate predictors chosen in previous approaches mentioned [16], [17] are not physiologically limiting for the native range borders of these species.

The striking question arising is genetic novelty (niche evolution) versus a better insight into the existing fundamental niche breadth. We claim that this should be more properly addressed when applying SDMs. Some authors have argued that SDM approaches using observed distributions for model computation per se rather reflect the realized than fundamental niche [18]. That may per se cause errors when projecting SDMs into new areas, since suitable areas may be excluded although being physiologically suitable for the target species. Modeling should thus focus on the physiological limits of species for maximum predictions. Without this information, many of the observed mismatches (or ‘niche shifts’) might simply be artifacts caused by a choice of unsuitable predictors. We hypothesize that a selection of predictors aiming at a description or even at a complete depiction of the climatic conditions in the native range may be less useful for statistical model training than predictor selections based on a mechanistic understanding of physiologically limiting factors.

So far, only a few studies have tried to model the fundamental niche of a species without using distribution records. In a comprehensive study physiological measurements of the Australian gecko *Heteronotia binoei* were combined with high-resolution climatic data to calculate the species' climate envelope and to project it into geographic space [19]. A similar study was undertaken on Cane toads (*Rhinella marina*) in Australia where it is an alien invasive species [20]. Such mechanistic approaches, with no doubt, are superior to the commonly used empirical methods. However, detailed information on the physiology and natural history traits required to fully address the fundamental niches from a mechanistic point of view will remain unavailable for most of the species on our planet. At least the predictors with physiological relevance are known in some species. Accordingly, empirical records and statistical models will remain a starting point, with, as we hypothesize, predictor sets based on natural history providing the most successful calibrations.

In order to test this hypothesis, the Slider turtle (*Trachemys scripta* Schoepff) may be a suitable study organism. It is an alien invasive species in many parts of the world and its ecology has been thoroughly studied. Between 1989 and 1997, about 52 million individuals were produced in the United States for the foreign pet trade [21]. Released by pet owners, it has established feral populations in many different regions of the world [22]–[25]; see IUCN Invasive Species Specialist Group (http://www.issg.org, search for ‘*Trachemys scripta elegans*’). At the same time, the natural history (including thermal tolerance, reproduction and physiology) of the Slider turtle has been the object of numerous studies [26]–[37], providing the basis for a natural history-driven modeling approach.

## Materials and Methods

### Slider Turtle Record Data

We used 375 Slider turtle records available through the Global Biodiversity Information Facility, GBIF (http://www.gbif.org) and HerpNet databases (http://www.herpnet.org) within the native range of the species, as defined in USGS Nonindigenous Aquatic Species Database (http://nas.er.usgs.gov/queries/FactSheet.asp?speciesID=1259). In addition, 205 records of invasive populations were obtained from the USGS Nonindigenous Aquatic Species Database (http://nas.er.usgs.gov/queries/FactSheet.asp?speciesID=1259), the Delivering Alien Invasive Species Inventories for Europe database, DAISE (http://www.europe-aliens.org), the IUCN Invasive Species Specialist Group (http.//www.issg.org), the Brazilian Instituto Hórus (http://www.instutohorus.org.br) and additional published references (Text S1). For georeferencing we used the Alexandria Digital Library Gazetteer Server Client (http://www.middleware.alexandria.ucsb.edu/client/gaz/adl/index.jsp). Accuracy of coordinates processed by us was assessed with DIVA-GIS [38]. In doing so, we only included invasive records with confirmed successful reproduction [22].

### Climate Data

**Figure 2:**
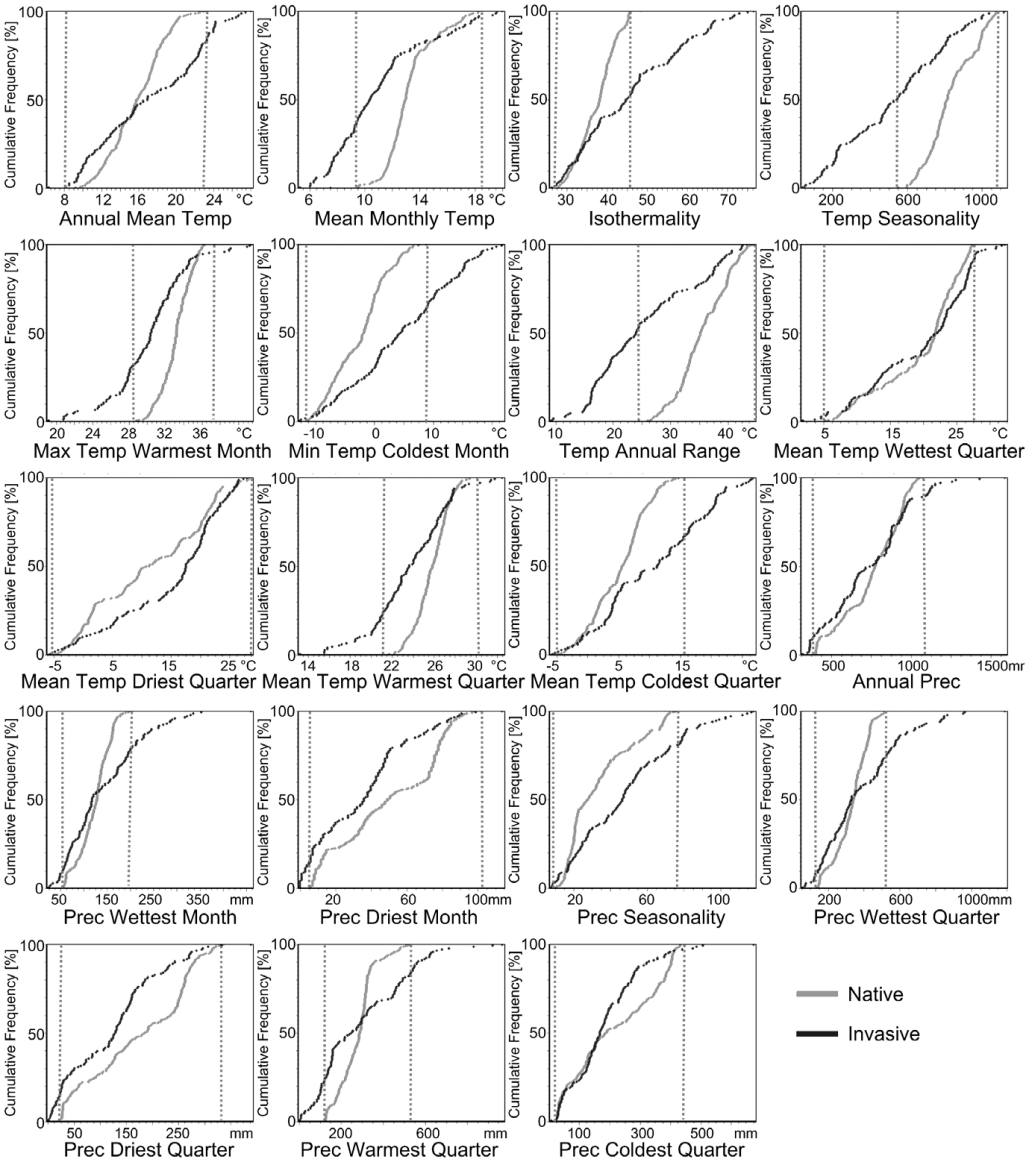
Comparison of 19 ‘bioclimate’ predictors at native and invasive records of the Slider turtle. Ranges of variables within the native records are indicated with vertical dashed lines. Note that some upper and lower limits of both native and invasive records are highly congruent.

Our climate information stems from WorldClim 1.4 [39], which is based on climate conditions in the period 1950–2000 at a spatial resolution of about 1×1 km. It was created by interpolation using a thin-plate smoothing spline of observed climate at weather stations, with latitude, longitude and elevation as independent variables (http://fennerschool.anu.edu.au/publications/software/). The climate data set was downloaded from the DIVA-GIS homepage (http://www.diva-gis.org), i.e. 36 monthly mean variables (minimum temperature, maximum temperature and precipitation). Based on these data, we calculated 19 ‘bioclimate’ variables for further processing with DIVA-GIS 5.4 [38]; see Figure 2 and Table S1. DIVA-GIS provide the opportunity to plot the cumulative frequency of distribution records according to ‘bioclimate’ variables. This allowed us to compare the climatic tolerance between the native and invasive distributions of the Slider turtle for all 19 ‘bioclimate’ variables.

### Selection of Climate Predictors

We chose three sets of variables as predictors for building SDMs: ‘comprehensive’ set: all 19 ‘bioclimate’ layers depicting the most comprehensive climatic pattern following the approach of different authors running SDM [6], [16]; ‘minimalistic’ set: a subset of seven variables out of the ‘comprehensive’ data set defining the availability of thermal energy and water (e.g. the minimum, maximum and mean values at the species records) as applied to different taxa [17], [40]–[42]; ‘natural history’ set: a subset of five variables out of the ‘comprehensive’ set aiming at reflecting the physiological limits of the Slider turtle's climate envelope (see Results). To be clear, we did not use these variables to map the known physiological limits. The variables were used as predictors in the same way as the other sets. In addition, we analyzed each 100 random subsets of seven and five ‘bioclimate’ variables, respectively, to test the null hypotheses that our selected variable sets ‘minimalistic’ and ‘natural history’ do not predict the potential distribution of invasive populations better than any random set consisting of the same number of variables. All sets, including the ‘minimalistic’ and ‘natural history’ sets, were extracted from the same set of WorldClim variables.

The Slider turtle strongly depends on continuous availability of water throughout the year, whereby almost any kind of water body is suitable (http://nas.er.usgs.gov/queries/FactSheet.asp?speciesID=1259). Therefore, it is not surprising that the south-western limit of its native range border is found in the North American deserts, which are characterized by low precipitation throughout the year (http://nas.er.usgs.gov/queries/FactSheet.asp?speciesID=1259). To take this into account, we included the ‘annual precipitation’ and the ‘precipitation of the driest quarter’ in our data set for SDMs. It has been demonstrated that the feeding behavior and digestive turnover rates in the Slider turtle are strongly temperature-dependent. At body temperature <10°C the species does not feed anymore [25], [43]. Hence, in accordance with a positive energetic balance over the year, we added the ‘annual mean temperature’ into our modeling approaches. The physiologically determined minimum equates with the minimum value recorded within the native range (8.3°C; see Table S1). It has been shown that the upper avoidance temperature is around 37°C [32] which is remarkably similar to the upper limit of the ‘maximum temperature of the warmest month’ recorded within the native range (i.e. 37.4°C; see Table S1). To account for this we included the ‘maximum temperature of the warmest month’ in SDM approaches.

Adult Slider turtles commonly hibernate at the bottom of icebound water bodies being largely insulated against cold air. They maintain a body temperature of approximately 4°C, which makes the species insensitive to cold winters. Nevertheless, Slider turtle records from Illinois were compared to those from eastern Iowa with contours identifying locations where frost penetrates to a depth of 12 cm in 11 out of 14 winters and found a strong relationship [34]. In colder parts of the native range, Slider turtle neonates hibernate inside their nests and are sensitive to temperatures below −0.6°C, at which they die; see also [36]. As a consequence, adult Slider turtles hibernating in water may tolerate frost, but neonates in nests may be negatively affected by frost. The native range of our study species to the north is therefore reasonably defined by minimum temperatures during winter. Considering this relationship, we included ‘minimum temperature of the coldest month’ when computing SDMs.

### Computation of SDMs

For the SDM building we used Maxent 3.2.19 [44] (http://www.cs.princeton.edu/~shapire/maxent), a machine-learning algorithm following the principles of maximum entropy. It has been shown to reveal better SDM results than other comparable methods [2], [45], [46]. A disadvantage of Maxent is that it is a ‘black box’ method. Since results can remarkably vary between different algorithms, we compared Maxent results with those obtained from a second algorithm BIOCLIM [47], [48], as implemented in DIVA-GIS. BIOCLIM develops SDMs by intersecting the ranges inhabited by the species along each environmental axis. An advantage of this method is that results are completely transparent for interpretation.

Clumped records can violate the statistical independence of observations and therefore assumptions of SDMs [49]. To account for this we extracted all ‘bioclimate’ values from the native distribution records and performed a cluster analysis with XLSTAT 2008 of Addinsoft (http://www.xlstat.com) in order to remove redundant information in the data set. XLSTAT allows to blunt cluster classes at a predefined threshold of similarity (herein 99.9%), and calculates mean values for each resulting class. These class means were used for further processing in SDMs.

DIVA-GIS allows for model testing by calculation of the Area Under the Curve (AUC), referring to the Receiver Operation Characteristic (ROC) curve by using a subset of data (commonly 25–30%) as test points and the remaining ones as training points [45], [50]. Independent validation (i.e. with invasive records) was suggested to be superior to data splitting [2]; therefore, we used all invasive Slider turtle records as subsets and in a second run 25% of the native records. Because absence data are lacking, DIVA-GIS uses a set of random pseudo-absence points [38]. AUC calculation is recommended for ecological applications because it is non-parametric. Values of AUC range from 0.5 for models with no predictive ability to 1.0 for models giving perfect predictions and, according to a given classification [51], AUC values >0.9 describe ‘very good’, >0.8 ‘good’ and >0.7 ‘useable’ discrimination ability. However, the reliability of AUC validation in ecological modeling has recently been questioned [52]. AUC values depend on the predicted degree of sites occupied by a species within the study area (i.e. AUC values for models describing the potential distribution of generalists are commonly lower than those computed for specialists). Nevertheless, relative comparisons of AUC values are useful for comparisons within areas of the same extend and when applying the same set of random background points.

For thresholds derived from the natural history and physiological traits describing the climate envelope of the Slider turtle, it is important to reduce the contribution of variables to their upper or lower tails, respectively. This is reasonable considering the limiting function of the ‘minimum temperature of the coldest month’, which may kill neonates. Here, only the lower tail has a biological meaning, but warmer temperatures may provide no disadvantage for the species. In BIOCLIM this kind of function is implemented directly, but is unfortunately absent in Maxent. Therefore, we used grids of each variable containing categorical classes between the upper or lower limits and the mean of the variables within the native range of the Slider turtle for Maxent runs. For parts of a grid representing the biologically meaningless tail, values greater or smaller than the mean of the variable within the native range were combined into a single category. These procedures remove the influence of meaningless tails during Maxent runs.

The logistic output of Maxent is a continuous map which allows fine distinctions to be made between the modeled suitability of different areas. Maxent calculates a threshold value at each run [44]. Values greater than this threshold may be interpreted as reasonable approximation of a species' potential distribution, but the higher a Maxent value, the better the prediction and therefore the climatic suitability for a species.

Six types of areas are mapped in the BIOCLIM output: areas outside the 0–100 percentile climatic envelope of the species for one or more ‘bioclimate’ variables are considered unsuitable, grid cells within the 0–2.5 percentile have a ‘low’ climatic suitability, those within the 2.5–5 percentile a ‘medium’, those within the 5–10 percentile a ‘high’, those within the 10–20 percentile a ‘very high’ and cells within the 20–100 percentile an ‘excellent’ climatic suitability [38].

## Results

Figure 2 compares each of the 19 ‘bioclimate’ variables of the native and invasive ranges of the Slider turtle, respectively. Ranges of variables observed in invasive populations which exceed those observed in native ones can be interpreted as shifts in niche dimension. Ranges in the following variables were most similar in native and invasive ranges: ‘annual mean temperature’, ‘mean temperature of the wettest quarter’, ‘mean temperature of the driest quarter’, ‘annual precipitation’, ‘precipitation of the driest month’, ‘precipitation of the driest quarter’ and ‘precipitation of the coldest quarter’. The highest dissimilarity was found in ‘isothermality’, ‘temperature seasonality’, ‘annual temperature range’, ‘minimum temperature of the coldest month’ and ‘mean temperature of the coldest quarter’. Lower temperature limits in the native and invasive ranges were almost equal for ‘annual mean temperature’, ‘isothermality’, ‘minimum temperature of the coldest month’, ‘mean temperature of the wettest quarter’ and ‘mean temperature of the driest quarter’, but the upper limits within the invasive range frequently exceeded those of the native range.

**Figure 3:**
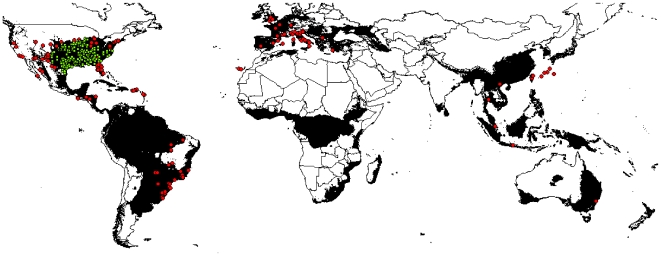
Worldwide occurrence of the Slider turtle. Shown is presence in the native range (green dots) and in the invasive range where the Slider turtle is known to reproduce (red dots) whereas areas considered as suitable to the species with respect to physiological limits as described in the text are indicated in black.

Areas meeting all climatic requirements of the species according to the expected physiological limits of the Slider turtle are mapped in Figure 3. Areas where any of the proposed climatic variables are outside the physiological limit of the species were excluded. The remaining area is highly coincident with the native range as well as records of native and invasive populations (AUC_native_ = 0.849; AUC_invasive_ = 0.795).

**Figure 4:**
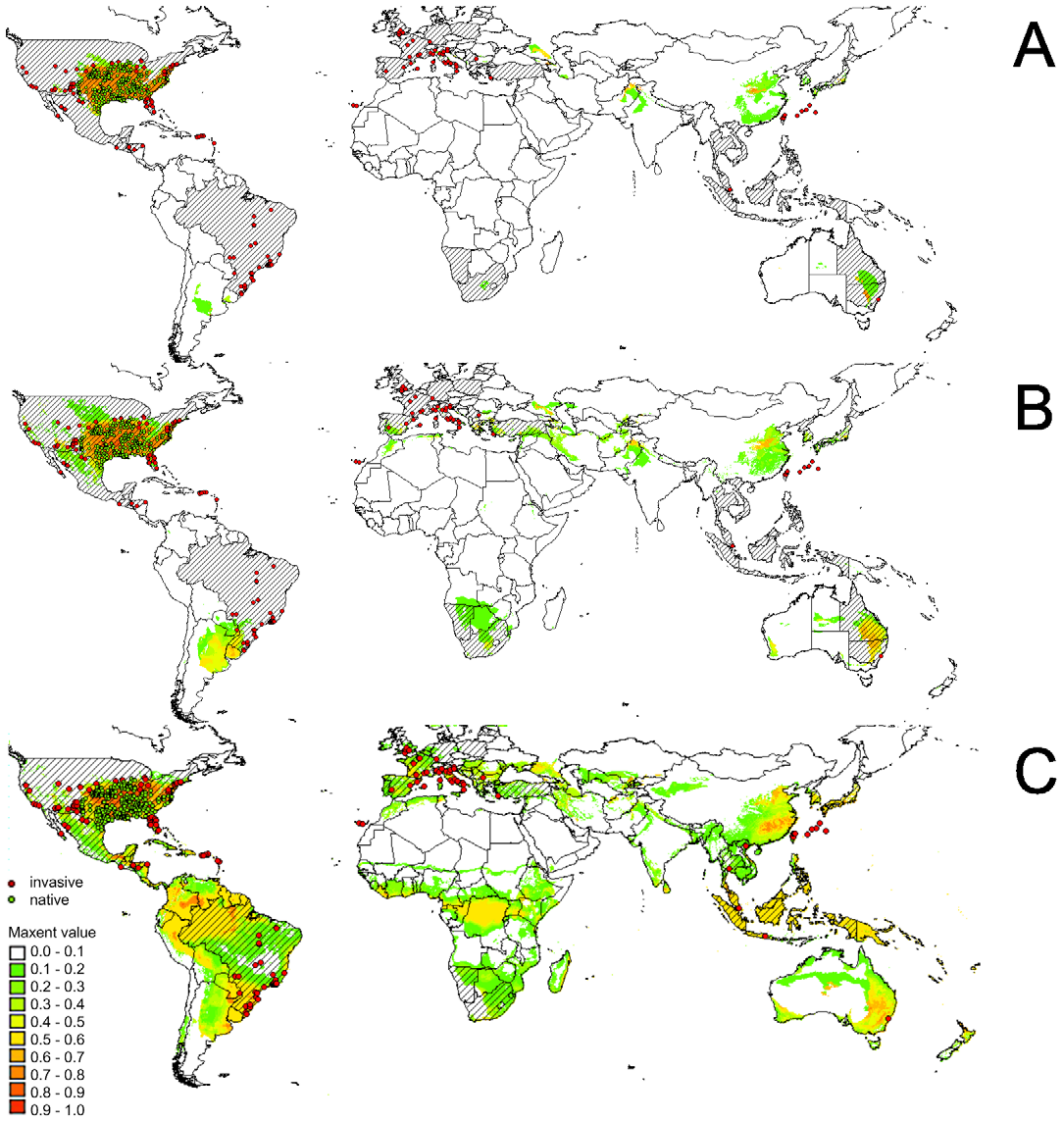
Species Distribution Model for the Slider turtle. Presence in the native (green dots) and invasive ranges where it is known to reproduce (red dots) are given. Countries from which reproducing populations of the Slider turtle are known but no specific localities are available are hatched and potential distribution derived from Maxent SDM is colored: (A) using 19 ‘bioclimate’ variables, approach ‘comprehensive’; (B) using 7 ‘bioclimate’ variables, approach ‘minimum’; (C) using 5 ‘bioclimate’ variables derived from physiological and natural history traits of the Slider turtle, approach ‘natural history’.

Applying the ‘comprehensive set’ of ‘bioclimate’ variables to SDM calculation predicted the native range in a way which matched the known natural distribution of the Slider turtle in both Maxent and BIOCLIM models. However, the models largely failed to predict populations elsewhere in the world due to overfitting (Figure 4A, see also Figure S1; Maxent AUC_native_ = 0.991; AUC_invasive_ = 0.716; BIOCLIM AUC_native_ = 0.990; AUC_invasive_ = 0.547). Using the ‘minimalistic’ subset of ‘bioclimate’ variables, SDM accuracy within the native range was reasonably met. However, predictions for invasion of the Slider turtle outside North America remained poor (Figure 4B; see also Figure S1; Maxent AUC_native_ = 0.989; AUC_invasive_ = 0.702; BIOCLIM AUC_native_ = 0.988; AUC_invasive_ = 0.535). In contrast, only the results for the ‘natural histroy’ subset of variables met both native and invasive potential distributions of the Slider turtle (Figure 4C; see also Figure S1; Maxent AUC_native_ = 0.974; AUC_invasive_ = 0.861; BIOCLIM AUC_native_ = 0.974; AUC_invasive_ = 0.757).

**Figure 5:**
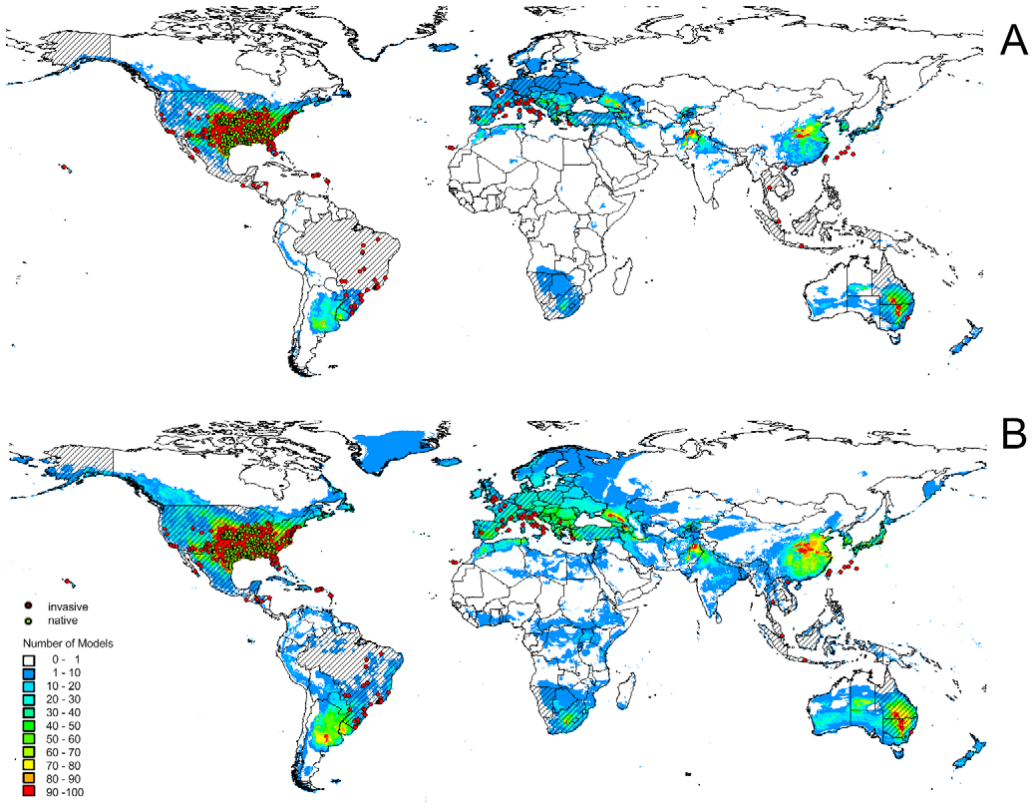
Prediction accuracy of models calculated. One-hundred summed Maxent models for the Slider turtle converted to presence/absence maps each calculated with a random selection of seven (A) and five (B) variables out of the complete set of 19 ‘bioclimate’ predictors. Note that the species' native range is well captured by all models whereby the invasive populations are not, especially between 26° N and S longitude.

**Figure 6:**
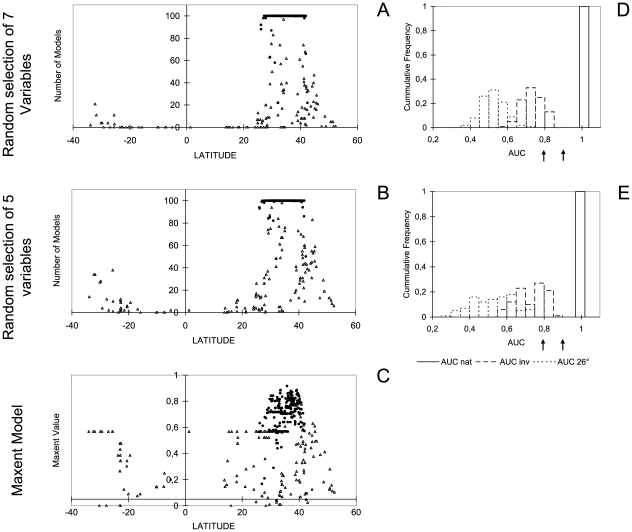
Predictive performance for invasive populations. It largely decrease at lower latitudes in 100 models computed with random subsets of predictors (A, B; the number of models predicting each record is indicated), but not in a Maxent model derived from natural history criteria (C; Maxent values per record is indicated) (filled dots: native records; open triangles: invasive records). Test statistics of 100 Maxent models based on random selection of each seven (D) and five variables (E) out of the complete set of 19 ‘bioclimate’ variables. Model accuracy was tested with native (AUC nat), invasive (AUC inv) and ‘tropical’ invasive records (AUC 26° N and S; N = 62). Note that the native range is well captured by each model whereby the invasive populations, especially in the tropics, are not. Arrows indicate AUC inv values of the Maxent model derived from natural history criteria (AUC inv = 0.861; AUC 26° N and S = 0.795).

The randomly selected subsets of five and seven ‘bioclimate’ variables revealed that all models were ‘very good’ in describing the native range (AUC _seven variables_ 0.987–0.994; AUC _five variables_ 0.977–0.994; Figure 5, Figure 6), which is slightly better then our models derived from the ‘natural history’ set. Comparing the predictive performance of the models outside the Slider turtle's native range, selection of a lower number of variables was associated with a broader area classified as suitable in a limited number of models (<10%). The AUC value of our model for invasive records derived from natural history criteria was higher than all AUC values obtained from random variable selection confirming a better prediction ability (AUC ranges seven random variables: native: 0.987–0.994, invasive: 0.587–0.847, AUC ranges five random variables: native: 0.977–0.994, invasive: 0.569–0.855; AUC data set ‘natural history’  = 0.861). In both random iterations, invasive records were less frequently captured than invasive records at the same latitudes as the native records (Figure 6A, B). This applies especially to records situated at lower latitudes (between 26° N and S corresponding to the southernmost known native records). This latitudinal decrease in predictive performance was confirmed when testing the models using only invasive records between 26° N and S as test points (N = 62; Figure 6D, E; AUC range seven random variables: 0.356–0.708; AUC range five random variables: 0.279–0.749), whereas our model derived from natural history criteria performed well here (Figure 6C, AUC = 0.795). Thus, the vast majority of models did not capture the Slider turtle's actual climate envelope.

## Discussion

Our results provide evidence that the observation of an apparent ‘niche’ (i.e. climate envelope) shift in the Slider turtle strongly depends on the choice of the variables applied during modeling. The observed range of a species reflects multiple determinants, including climatic tolerances, biotic interactions, equilibrium with climate and dispersal limitation. Hence, niche-based models derived from distribution alone will predict the geographic equivalent of the realized niche rather than the potential range of a species [18]. A SDM derived from the realized niche may therefore under-predict a species' fundamental niche because it does not consider biotic interactions and abiotic factors which may limit distributions. In our study species, one such abiotic factor is probably the ocean, which limits the native range south- and eastwards. This illustrates that not all range limits can be explained by climate alone what strongly affects the models herein by frequently excluding areas between 26° N and S.

Furthermore, when applying a data set depicting the complete climatic variation within the realized distribution of a species, the limits of all dimensions of its fundamental niche are unlikely to be reached because some niche dimensions may have a wide-reaching impact defining a large part of the native range border (as the ‘minimum temperature of the coldest month’ in the Slider turtle). Likewise, others may have no impact. However, the parameters without an actual limiting function could be treated as limiting in SDM and may exclude areas suitable for the target species outside the native range from a natural history point of view.

Although the SDM approach may provide insight into the fundamental niche of a species [3], [7], it cannot provide a complete picture and might be poor in choosing the relevant determinants of distribution patterns. Our results imply that parameters, which are unrelated to a species' natural history and physiology albeit congruent with its range limits, are frequently used by the models as proxies for a species' climatic envelope. This becomes evident in comparing the predictive performance of our models in the invaded range computed with a random selection of variables and a model derived from natural history criteria (Figure 5A, B). Only the model considering explicit natural history traits performed significantly better than models based on an equal number of randomly chosen variables (Figure 4C, Figure 6C; see also Figure S1). The vast majority of random models did not capture the Slider turtle's actual climate envelope although test statistics may suggest a reasonably high model quality. Hence, the observed mismatches may be misinterpreted as range shifts rather than as errors in the selection of variable (Figure 4A, B, Figure 5A, B; see also Figure S1). It was found that the predictive power of models in respect of native and introduced distributions is strongly affected by the different environmental data sets applied [53]. These findings are congruent with our results, since different sets of predictor variables have a different chance of capturing a greater or smaller part of the niche dimensions restricting a species' native range, thus explaining their different prediction success.

Assuming a shift in the slider turtle's fundamental niche is not necessary to explain the range of invasive populations in SDM, as mismatches between climate envelopes in native and invasive ranges can simply be explained by the choice of variables in SDM. Before any conclusions on niche shifts are made, an assessment of a species' fundamental niche should be addressed based on a mechanistic understanding of the limiting factors of its range. Our results indicate that such an understanding of causal factors is essential when assessing the climatic suitability of a geographic area or potential range shifts in past or future scenarios.

Our study does not aim at a principle rejection of a fundamental niche shift occurring during invasion processes [16], [17], [54]. If in fact a niche shift had occurred in invasive populations of the Slider turtle our conclusions will be based on the false assumption (i.e. no niche shift). However, we are convinced that assuming inappropriate model selection instead of niche shift (evolutionary response) in a successful invader is a more parsimonious assumption, especially in the light of all the methodological uncertainties accompanying with SDMs [45]. First, within 30 years no more than two Slider turtle generations may have occurred which makes evolutionary change unlikely. Second, over this time span the species has conquered different parts of the world, so evolutionary change should have taken place multiple times. These observations raise some concerns about the simplistic approach of applying ‘standard datasets’ of predictors in climate envelope modeling.

In conclusion, the mismatch between ‘very good’ [51] model performance in a mere statistical sense and the model's ability to capture the climatic niche of an organism is of particular concern. Selection of variables must be conducted carefully and needs to be fitted to the ecological and physiological characteristics of each species. Unfortunately, the lack of physiological data for the vast majority of species and the application of ‘standard’ sets of environmental variables make predictions for whole species' communities and biodiversity loss questionable [55], [56]. Thus, future research should place more emphasis on the evaluation of the physiological and ecological important characteristics which are important for each single species instead of being content with deductions from distributional information.

## Supporting Information

Figure S1 Presence of the Slider turtle in its native range (green dots) and invasive range where it is known to reproduce (red dots), countries from which reproducing populations are known but no specific localities are available (hatched) and potential distribution derived from BIOCLIM SDM (colored): (A) using 19 ‘bioclimate’ variables, approach ‘comprehensive’; (B) using 7 ‘bioclimate’ variables, approach ‘minimalistic’; (C) using 5 ‘bioclimate’ variables derived from physiological and natural history traits of the Slider turtle, approach ‘natural history’.
(2.13 MB DOC)

Table S1 Variation of 19 ‘bioclimate’ variables within the native and invasive ranges of the Slider turtle.
(0.06 MB DOC)

Text S1 References used for Slider turtle records.
(0.03 MB DOC)

## References

[1] Lowe S, Browne M, Boudjelas S, De Poorter M (2000). 100 of the world's worst invasive alien species. A selection from the Global Invasive Species Database. Auckland, CA: The IUCN Invasive Species Specialist Group (ISSG). 12 p..

[2] Jeschke JM, Strayer DL (2008). Usefulness of bioclimatic models for studying climate change and invasive species.. Ann N Y Acad Sci.

[3] Peterson AT (2001). Predicting species' geographic distributions based on ecological niche modeling.. Condor.

[4] Peterson AT (2003). Predicting the geography of species' invasions via ecological niche modeling.. Q Rev Biol.

[5] Rödder D, Schmidtlein S, Schick S, Lötters S, Hodkinson T, Jones MB, Waldren S, Parnell JAN (2009). Climate envelope models in systematics and evolutionary research: theory and practice.. Climate change, ecology and systematics.

[6] Giovanelli JGR, Haddad CFB, Alexandrino J (2007). Predicting the potential distribution of the alien invasive American bullfrog (*Lithobates catesbeianus*) in Brazil.. Biol Invas.

[7] Peterson AT, Vieglais DA (2001). Predicting species invasions using ecological niche modeling: new approaches from bioinformatics attack a pressing problem.. BioSci.

[8] Rödder D, Solé M, Böhme W (2008). Predicting the potential distribution of two alien invasive Housegeckos (Gekkonidae: *Hemidactylus frenatus*, *Hemidactylus mabouia*).. North-West J Zool.

[9] Rödder D (2009). ‘Sleepless in Hawaii’ – does anthropogenic climate change enhance ecological and socioeconomic impacts of the alien invasive *Eleutherodactylus coqui* Thomas, 1966 (Anura: Eleutherodactylidae)?. North-West J Zool.

[10] Welk E, Schubert K, Hoffman MH (2002). Present and potential distribution of invasive garlic mustard (*Alliaria petiolata*) in North America.. Divers Distrib.

[11] Soberón J, Peterson AT (2005). Interpretation of models of fundamental ecological niches and species' distributional areas.. Biodiv Inform.

[12] Hutchinson GE (1957). Concluding Remarks.. Cold Spring Harbor Symposia on Quant Biol.

[13] Hutchinson GE (1978). An introduction to population ecology..

[14] Pearman PB, Guisan A, Broennimann O, Randin CF (2008). Niche dynamics in space and time.. TREE.

[15] Sax DF, Stachowicz JJ, Brown JH, Bruno JF, Dawson MN (2008). Ecological and evolutionary insights from species invasions.. TREE.

[16] Broennimann O, Treier UA, Müller-Schärer H, Thuiller W, Peterson AT (2007). Evidence of climatic niche shift during biological invasion.. Ecol Let.

[17] Fitzpatrick MC, Weltzin JF, Sanders NJ, Dunn RR (2007). The biogeography of prediction error: why does the introduced range of the fire ant over-predict its native range?. Glob Ecol Biogeogr.

[18] Pulliam HR (2000). On the relationship between niche and distribution.. Ecol Let.

[19] Kearney M, Porter WP (2004). Mapping the fundamental niche: physiology, climate, and the distribution of a nocturnal lizard.. Ecol.

[20] Kearney M, Phillips BL, Tracy CR, Christian KA, Betts G (2008). Modeling species distributions without using species distributions: the cane toad in Australia under current and future climates.. Ecography.

[21] Telecky TM (2001). United States import and export of live turtles and tortoises.. Turtle Tortoise Newsl.

[22] Ficetola GF, Thuiller W, Padoa-Schioppa E (2009). From introduction to the establishment of alien species: bioclimatic differences between presence and reproduction localities in the slider turtle.. Divers Distrib.

[23] Ota H, Toda M, Masunaga G, Klkukawa A, Toda M (2004). Feral populations of amphibians and reptiles in the Ryukyu Archipelago, Japan.. Glob Environ Res.

[24] Perry G, Owen JL, Petrovic C, Lazell J, Egelhoff J (2007). The Red-eared slider, *Trachemys scripta elegans*, in the British Virgin Islands.. Appl Herpetol.

[25] Ramsay NF, Ng PKA, O'Riordan M, Ming Chou L, Gherardi F (2007). The Red-eared slider (*Trachemys scripta elegans*) in Asia: a review.. Biological invaders in inland waters: profiles, distribution, and threats.

[26] Crews D, Bergeron JM, Bull JJ, Flores D, Touisignant A (1994). Temperature-dependent sex determination in reptiles: proximate mechanisms, ultimate outcomes, and practical applications.. Dev Genet.

[27] Baccari GC, Minucci S, Dimatteo L (1993). The orbital glands of the terrapin *Pseudemys scripta* in response to osmotic stress - A light and electron microscope study.. J Anat.

[28] Bailey JR, Driedzic WR (1995). Short-term anoxia does not impair protein turnover in isolated perfused turtle heart.. J Comp Physiol B, Biochem Syst Environ Physiol.

[29] Garstka WR, Cooper WE, Wasmund KW, Lovich JE (1991). Male sex steroids and hormonal control of male courtship behavior in the Yellow-bellied slider turtle, *Trachemys scripta*.. Comp Biochem Physiol.

[30] Hutchison VH, Vinegar A, Kosh RJ (1966). Critical thermal maxima in turtles.. Herpetologica.

[31] Hutchison VH, Harless M, Morlock H (1979). Thermoregulation.. Turtles: perspectives and research.

[32] Lamb T, Bickham JW, Lyne TB, Gibbons JW (1995). The Slider turtle as an environmental sentinel: Multiple tissue assays using flow cytometric analysis.. Ecotoxicology.

[33] Packard GC, Trucker JK, Nicholson D, Packard MJ (1997). Cold tolerance in hatchling Slider turtles (*Trachemys scripta*).. Copeia 1997.

[34] Rödder D, Kwet A, Lötters S (2009). : Translating natural history into geographic space: a macroecological perspective on the North American Slider, *Trachemys scripta* (Reptilia, Cryptodira, Emydidae).. J Nat Hist.

[35] Tucker JK, Filoramo NI, Paukstis GL, Janzen FJ (1998). Response of Red-eared Slider, *Trachemys scripta elegans*, eggs to slightly differing water potentials.. J Herpetol.

[36] Tucker JK, Packard GC (1998). Overwinter survival by hatchling Sliders (*Trachemys scripta*) in West-Central Illinois.. J Herpetol.

[37] Wibbels T, Bull JJ, Crews D (1991). Chronology and morphology of temperature-dependent sex determination.. J Exp Zool.

[38] Hijmans RJ, Cruz JM, Rojas E, Guarino L (2001). DIVA-GIS, version 1.4. A geographic information system for the management and analysis of genetic resources data..

[39] Hijmans RJ, Cameron SE, Parra JL, Jones PG, Jarvis A (2005). Very high resolution interpolated climate surfaces for global land areas.. Intern J Climatol.

[40] Hijmans RJ, Graham CH (2006). The ability of climate envelope models to predict the effect of climate change on species distributions.. Glob Chang Biol.

[41] Peterson AT, Nyári ÁS (2007). Ecological niche conservatism and pleistocene refugia in the Thrush-like Mourner, *Shifforinis* sp., in the Neotropics.. Evol.

[42] Ficetola GF, Thuiller W, Miaud C (2007). Prediction and validation of the potential global distribution of a problematic alien species - the American bullfrog.. Divers Distrib.

[43] Parmenter RR (1980). Effects of food availability and water temperature on the feeding ecology of Pond sliders (*Chrysemys s. scripta*).. Copeia 1980.

[44] Phillips SJ, Anderson RP, Schapire RE (2006). Maximum entropy modeling of species geographic distributions.. Ecol Model.

[45] Elith J, Graham CH, Anderson RP, Dudík M, Ferrier S (2006). Novel methods improve prediction of species' distributions from occurrence data.. Ecography.

[46] Wisz MS, Hijmanss RJ, Peterson AT, Graham CH, Guisan A (2008). Effects of sample size on the performance of species distribution models.. Divers Distrib.

[47] Nix H, Longmore R (1986). A biogeographic analysis of Australian elapid snakes.. Atlas of elapid snakes of Australia.

[48] Busby JR, Margules CR, Austin MP (1991). BIOCLIM - a bioclimatic analysis and prediction system.. Nature conservation: cost effective biological surveys and data analysis.

[49] Dormann CF, McPherson J, Araújo MB, Bivand R, Bolliger J (2007). Methods to account for spatial autocorrelation in the analysis of species distributional data: a review.. Ecography.

[50] Pearce J, Ferrier S (2000). Evaluating the predictive performance of habitat models developed using logistic regression.. Ecol Model.

[51] Swets K (1988). Measuring the accuracy of diagnostic systems.. Science.

[52] Lobo JM, Jiménez-Valverde A, Real R (2008). AUC: a misleading measure of the performance of predictive distribution models.. Glob Ecol Biogeogr.

[53] Peterson AT, Nakazawa Y (2008). Environmental data sets matter in ecological niche modeling: an example with *Solenopsis invicta* and *Solenopsis richteri*.. Glob Ecol Biogeogr.

[54] Holt RD, Barfield M, Gomulkiewicz R, Sax D, Stachowicz J, Gaines SD (2005). Theories of niche conservatism and evolution: could exotic species be potential tests?. Species invasions: insight into ecology, evolution, and biogeography.

[55] Malcom JR, Liu D, Neilson RP, Hansen L, Hannah L (2006). Global warming and extinction of endemic species from biodiversity hotspots.. Conserv Biol.

[56] Thomas CD, Cameron A, Green RE, Bakkenes M, Beaumont LJ (2004). Extinction risk from climate change.. Nature.

