# Supplementary material for: Alien Invasive Slider Turtle in Unpredicted Habitat: A Matter of Niche Shift or of Predictors Studied?
Source: PLoS One. 2009 Nov 24;4(11):e7843. doi: 10.1371/journal.pone.0007843 (PMC2776975; doi:10.1371/journal.pone.0007843)

**Figure S1**

Presence of the Slider turtle in its native range (green dots) and invasive range where it is known to reproduce (red dots), countries from which reproducing populations are known but no specific localities are available (hatched) and potential distribution derived from BIOCLIM SDM (colored): (A) using 19 ‘bioclimate’ variables, approach ‘comprehensive’; (B) using 7 ‘bioclimate’ variables, approach ‘minimalistic’; (C) using 5 ‘bioclimate’ variables derived from physiological and natural history traits of the Slider turtle, approach ‘natural history’.


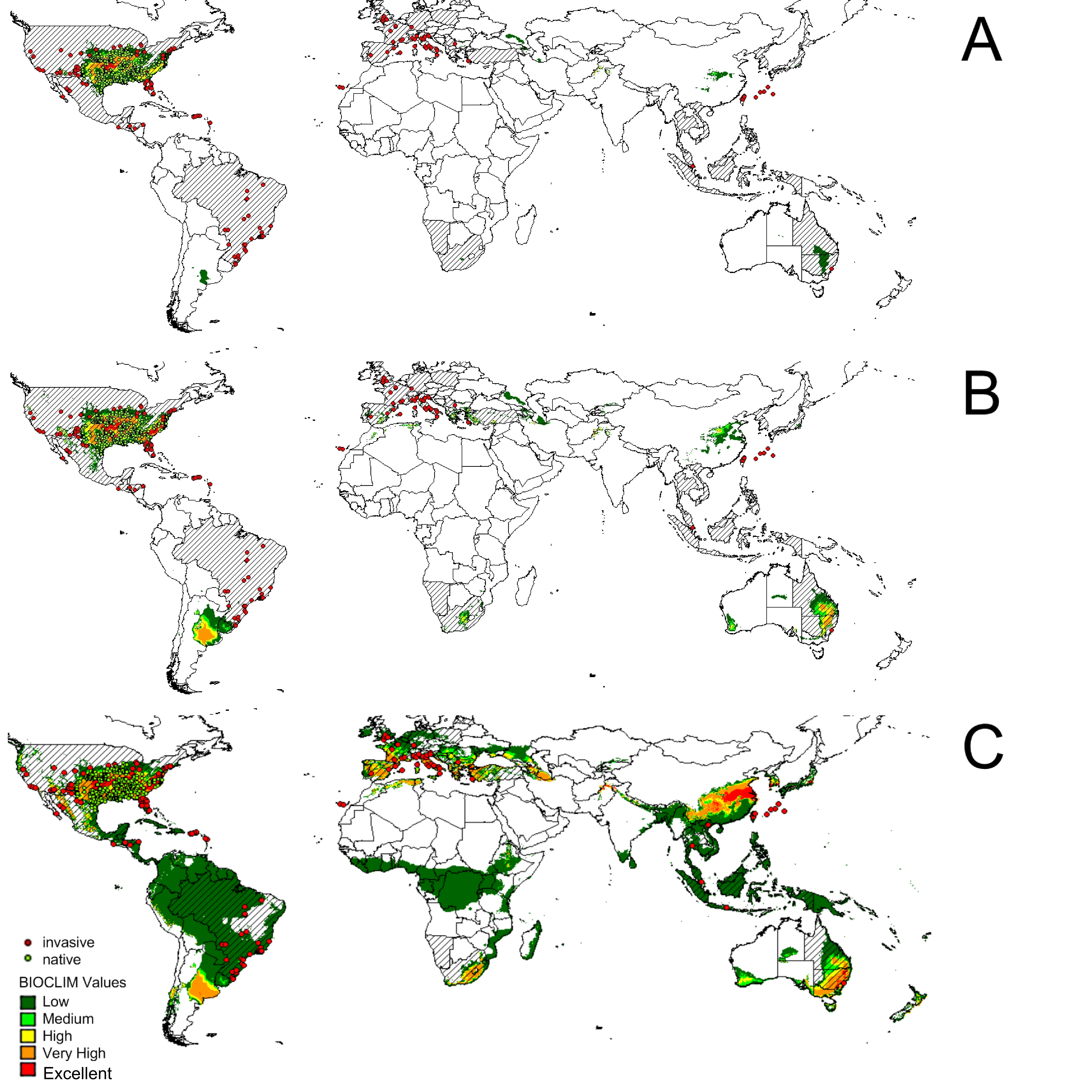

Supplement: Figure S1 — Presence of the Slider turtle in its native range (green dots) and invasive range where it is known to reproduce (red dots), countries from which reproducing populations are known but no specific localities are available (hatched) and potential distribution derived from BIOCLIM SDM (colored): (A) using 19 ‘bioclimate’ variables, approach ‘comprehensive’; (B) using 7 ‘bioclimate’ variables, approach ‘minimalistic’; (C) using 5 ‘bioclimate’ variables derived from physiological and natural history traits of the Slider turtle, approach ‘natural history’. (2.13 MB DOC) [file pone.0007843.s001.doc]
