# Supplementary material for: Alien Invasive Slider Turtle in Unpredicted Habitat: A Matter of Niche Shift or of Predictors Studied?
Source: PLoS One. 2009 Nov 24;4(11):e7843. doi: 10.1371/journal.pone.0007843 (PMC2776975; doi:10.1371/journal.pone.0007843)
Supplement: Table S1 — Variation of 19 ‘bioclimate’ variables within the native and invasive ranges of the Slider turtle. (0.06 MB DOC) [file pone.0007843.s002.doc]

**Table S1**

Variation of 19 ‘bioclimate’ variables within the native and invasive ranges of the Slider turtle.

|  | native records | | | |  | invasive Records | | | |
| --- | --- | --- | --- | --- | --- | --- | --- | --- | --- |
|  | min | mean | max | SD |  | min | mean | max | SD |
| annual mean temperature [°C] | 8.3 | 15.6 | 23.3 | 2.9 |  | 6.4 | 16.8 | 28.0 | 5.4 |
| mean monthly temperature [°C] | 7.6 | 13.4 | 18.6 | 1.8 |  | 5.3 | 11.5 | 20.0 | 3.8 |
| isothermality | 26.2 | 37.3 | 48.5 | 4.6 |  | 26.4 | 45.2 | 76.6 | 12.5 |
| temperature seasonality | 548.6 | 849.1 | 1095.4 | 130.0 |  | 41.0 | 564.5 | 1123.1 | 270.1 |
| maximum temperature warmest month [°C] | 28.9 | 33.3 | 37.4 | 1.6 |  | 19.2 | 30.5 | 41.4 | 4.0 |
| minimum temperature coldest month [°C] | -12.6 | -2.9 | 8.9 | 4.7 |  | -12.6 | 3.7 | 21.8 | 8.7 |
| temperature annual range [°C] | 24.6 | 36.2 | 44.5 | 4.5 |  | 9.3 | 26.8 | 43.4 | 9.2 |
| mean temperature wettest quarter [°C] | 5.3 | 20.0 | 27.9 | 5.7 |  | 1.9 | 19.8 | 32.3 | 7.1 |
| mean temperature driest quarter [°C] | -5.8 | 10.4 | 28.7 | 9.5 |  | -5.6 | 14.5 | 28.0 | 8.8 |
| mean temperature warmest quarter [°C] | 21.2 | 25.8 | 30.1 | 1.5 |  | 13.3 | 23.7 | 32.5 | 3.5 |
| mean temperature coldest quarter [°C] | -5.8 | 4.8 | 15.8 | 4.4 |  | -5.6 | 9.9 | 26.9 | 8.2 |
| annual precipitation [mm] | 278.0 | 989.6 | 1652.0 | 372.1 |  | 142.0 | 942.6 | 2682.0 | 521.4 |
| precipitation wettest month [mm] | 55.0 | 121.9 | 203.0 | 32.9 |  | 25.0 | 135.2 | 460.0 | 80.9 |
| precipitation driest month [mm] | 6.0 | 47.3 | 102.0 | 28.7 |  | 0.0 | 34.6 | 114.0 | 26.8 |
| precipitation seasonality | 9.9 | 35.6 | 77.3 | 18.3 |  | 7.9 | 48.1 | 118.3 | 27.1 |
| precipitation wettest quarter [mm] | 138.0 | 327.6 | 524.0 | 89.8 |  | 59.0 | 369.1 | 1190.0 | 221.8 |
| precipitation driest quarter [mm] | 22.0 | 164.6 | 337.0 | 96.1 |  | 2.0 | 121.2 | 389.0 | 87.9 |
| precipitation warmest quarter [mm] | 123.0 | 277.8 | 524.0 | 82.1 |  | 6.0 | 281.6 | 967.0 | 207.1 |
| precipitation coldest quarter [mm] | 22.0 | 202.6 | 449.0 | 139.0 |  | 9.0 | 176.7 | 674.0 | 116.2 |
