# Supplementary material for: Alien Invasive Slider Turtle in Unpredicted Habitat: A Matter of Niche Shift or of Predictors Studied?
Source: PLoS One. 2009 Nov 24;4(11):e7843. doi: 10.1371/journal.pone.0007843 (PMC2776975; doi:10.1371/journal.pone.0007843)
Supplement: Text S1 — References used for Slider turtle records. (0.03 MB DOC) [file pone.0007843.s003.doc]

**Text S1**

References used for Slider turtle records.

Aresco MJ, Jackson DR (2006) *Trachemys scripta elegans*. Herp Rev 37:239-240

Arvy C, Servan J (1998) Imminent competition between *Trachemys* *scripa* and *Emys* *orbicularis* in France. In: U. Fritz (ed) Proceedings of the EMYS symposium Dresden 96, DGHT, Rheinbach, Germany pp 33-40

Cadi A, Delmas V, Prévot-Julliard A-C, et al (2004) Successful reproduction of the introduced Slider turtle (*Trachemys scripta elegans*) in the Southern of France. Aqu Conserv: Marine Freshwater Ecosyst 14:237-246

Chen T-H (2006) Distribution and status of the introduced Red-eared slider (*Trachemys scripta elegans*) in Taiwan. In: Koike F, Clout MN, Kawarnichi M, De Poorter M, Iwatsuki K (ed) Assessment and control of biological invasion risk. Shoukadoh Book Sellers, Kyoto, Japan, and Gland, Switzerland, pp 187-195

Da Silva E, Blasco M (1995) *Trachemys scripta elegans* in southwestern Spain. Herp Rev 26:133-134

Degenhardt WG, Painter CW, Price AH (1996) Amphibians and reptiles of New Mexico. Albuquerque

Dixon JR (2000) Amphibians and reptiles of Texas. Second edition. Texas A and M University Press, College Station

Ehret DJ, Parker D (2005) Geographic distribution: *Trachemys scripta elegans* (Red-eared slider). Herp Rev 36:78

Emer S (2004) Growth of an introduced population of *Trachemys scripta elegans* at Fox Pond, Eckerd College, Pinellas country, Florida. Herp Rev 35:34-35

Ficetola GF, Monti A, Padoa-Schioppa E (2002) First record of reproduction of *Trachemys scripta* in the Po Delta. Ann Museo Civila St. Natureza Ferrara 5:125-128

Jones BK (1988) Distribution and habitat associations of herpetofauna in Arizona: comparisons by habitat type. In: Szaro RC, Severson KE, Patton DR (ed) Management of amphibians, reptiles, and small mammals in North America. Proceedings of the Symposium. July 19 - 21 1988. United States Department of Agriculture Forest Service, General Technical Report RM-166, Arizona p 458

Luiselli L, Capula M, Capizzi D, et al (1997) Problems for conservation of pond turtles (*Emys orbicularis*) in central Italy: is the introduced Red-Eared Turtle (*Trachemys scripta elegans*) a serious threat? Chelon Conserv Biol 2:417-419

Martínez-Silvestre A, Soler J, Solé R, et al (1997) Nota sobre la reprocucción en condiciones naturales de la tortuga de florida (*Trachemys scripta elegans*) en Masquefa, (Cataluna, Espana). Boletin De La Associacion Herpetologica Español 840-42

McKeown S (1996) A field guide to reptiles and amphibians from the Hawaiian islands. Diamond Head Publishing, Inc., Los Osos, CA

Minton SA (2001) Amphibians and reptiles of Indiana. Revised 2nd Edition. Indiana Academy of Science, Indianapolis

Novotny RJ (1997) Geographic distribution: *Trachemys scripta elegans*. Herp Rev 28:95

Pieh A, Laufer H (2006) Die Rotwangen-Schmuckschildkröte (*Trachemys scripta elegans*) in Baden-Württemberg - mit Hinweis auf eine Reproduktion im Freiland. Z Feldherpetol 13:225-234

Platt SG, Snyder WE (1996) Geographic distribution: *Trachemys scripta elegans*. Herp Rev 27:151

Prévot-Julliard A-C, Gousset E, Archinard E, et al (2007) Pets and invasion risk: is the Slider turtle strictly carnivorous? Amphibia-Reptilia 28:139-143

Saenz D, Collins CS (1999) Geographic distribution: *Trachemys scripta*. Herp Rev 30:109

Schwartz A, Henderson RW (1991) Amphibians and reptiles of the West Indies: descriptions, distributions, and natural history. University of Florida Press, Gainesville, FL

Stitt EW, Brown D, Balfour PS (2004) Geographic distribution: *Trachemys scripta* *elegans* (Red-eared slider). Herp Rev 35:187

Townsend J.H, Krysko KL, Reppas AT, Sheehy CM (2002) Noteworthy records for introduced reptiles and amphibians from Florida, USA. Herp Rev 33:75
